# HJP 272, an endothelin receptor antagonist, and its role in cancer cell migration and invasion

**DOI:** 10.1016/j.tranon.2025.102492

**Published:** 2025-08-05

**Authors:** Nabeela Baig, Rameswari Chilamakuri, Saurabh Agarwal, Aaron Muth, Sandra E. Reznik

**Affiliations:** a Department of Pharmaceutical Sciences, St. John’s University, Queens, NY 11439, USA; b Departments of Pathology and Obstetrics and Gynecology and Women’s Health, Albert Einstein College of Medicine, Bronx, NY 10461, USA

**Keywords:** HJP 272, endothelin axis, cancer metastasis

## Abstract

**Introduction:**

Endothelins (ETs) are a family of versatile peptides composed of 21 amino acids with three isoforms: ET-1, ET-2, and ET-3. As the most abundant of the three isoforms, ET-1 is involved in various biological processes, such as regulation of vascular tone, humoral homeostasis, and neural crest development. However, focus is now being directed towards investigating the functions of the ET axis in the progression of different tumor types including ovarian, prostate, breast, lungs etc. HJP 272 is a novel ET_A_R antagonist and while our group has previously researched its effects on lung inflammation and preterm birth, this study marks the first time its role in cancer has been explored.

**Methods:**

We evaluated the *in vitro* activities of HJP 272 in the ET-1 and ET_A_R overexpressing cell lines MDA-MB-231 (TNBC), and A549 (NSCLC). While HJP 272 had no effect on the viability of cancer cells, we observed a significant inhibition in the migration, invasion, and clonogenic capacities of both cell lines. RNA-seq and western blot data demonstrate the potential underlying molecular mechanisms of this compound *in vitro*. Furthermore, HJP 272 was evaluated in a 3D spheroid assay for its ability to inhibit tumor formation in both cell lines, revealing a significant change in MDA-MB-231 cells while no significant changes were observed in A549 cells.

**Conclusions:**

Our work indicates a therapeutic potential for HJP 272 in cancer metastasis. The distinct outcomes between the two cell lines shed light on the potential differences of HJP 272′s effects across multiple cancer types.

## Highlights

• HJP 272 inhibits migration, invasion and clonogenesis of A549 and MDA-MB-231 cells.
• HJP 272 decreases expression of CDK-Rb-E2F axis genes in A549 cells.
• HJP 272 decreases expression of IL-17 pathway genes in MDA-MB-231 cells.
• HJP 272 decreases expression of and extracellular matrix genes in MDA-MB-231 cells.
• HJP 272 inhibits MDA-MB-231 spheroid growth.

## Introduction

Endothelins (ETs) are a family of versatile peptides comprised of 21 amino acids which play crucial roles in biological processes such as the regulation of vascular tone, humoral homeostasis and neural crest development [[1], [2], [3], [4], [5]]. The discovery of ET-1 can be dated back to 1985, when a “factor” with vasoconstrictor activity was acquired from a culture media of bovine aortic endothelial cells [6]. ET-1 belongs to a family of three isoforms in mammals that include: ET-1, ET-2 and ET-3, each significantly different from the other in their affinities for the different ET receptor subtypes [[7], [8], [9], [10], [11]]. The structural commonality between the isoforms is the presence of disulphide bonds and a tryptophan C-terminal moiety [7]. While the ET system plays a role in various physiological and developmental processes in vertebrates, such as neurotransmission, wound healing, and kidney homeostasis [12], its broad role in pathologic processes makes it an excellent therapeutic target [13,14].

A prominent breakthrough came with the discovery of the vasoconstrictive properties of ET-1 by Yanagisawa et al. in 1988 [6]. Subsequently, two ET G-protein coupled receptors (GPCRs) were identified, ET_A_ (ET receptor A) and ET_B_ (ET receptor B) [7,15]. Differences in binding affinities exist among these receptor subtypes, where ET-1 and ET-2 have greater affinity for ET_A_ than ET_B_ while all three isoforms, ET-1, ET-2 and ET-3 have equal affinity towards ET_B_ [6,7,16]. ET_A_ receptors are primarily found in the smooth muscles of blood vessels and coronary arteries of the heart, and they are involved in regulating vasoconstriction. In contrast, ET_B_ receptors are found in the endothelium where they mediate vasodilation [17].

Of the three existing isoforms, research has been mainly focused on ET-1, since it is the most potent, abundant, and multifunctional of the three[1]. The three isoforms of ET, ET-1, ET-2, and ET-3 along with their respective GPCRs, namely ET_A_R and ET_B_R, as well as ECE-1 and ECE-2 constitute the ‘ET axis’ [18]. The ET axis controls several important physiological processes, including vascular growth, inflammation, and vascular tone while its role in cancer is associated with the development and progression of cancer, angiogenesis, apoptosis, invasion, migration, and drug resistance, highlighting its significance as a potential target in cancer treatment [19]. As a mitogen, ET-1 can interact with epidermal growth factors, platelet-derived growth factor (PDGF) and transforming growth factor in smooth muscle cells and fibroblasts, demonstrating a positive role in growth and inhibiting apoptosis, consistent with its role in neoplasia [20].

ET-1 promotes growth in both epithelial tumors and non-epithelial tumors such as melanoma [19]. The activation of kinases, such as protein kinase C (PKC), epidermal growth factor (EGF), insulin-like growth factor-1 and mitogen-activated kinase (MAPK), all involved in cell survival and proliferation, are set in motion by ET-1 [21]. Subsequently, ET-1 can also activate signaling pathways involved in tumor progression via β-arrestin [22]. Moreover, ET-1 is a driver of some of the hallmarks of cancer, including angiogenesis, epithelial-to-mesenchymal transition (EMT), invasion and metastasis [21,23,24]. Elevated expression of ET-1 has been reported in lung, prostate, liver, breast, colon, and ovarian cancer [[25], [26], [27], [28]].

Some of the pathways activated by ET-1 include MAPK, PI3K-AKT, ERK1/2, NF-κB, B-catenin, HIF1a, RHO, phospholipases [29,30]. Prior research has explored the role of the ET axis in various types of mammalian cancers, including colorectal cancer through stimulation of adjacent fibroblasts [31]. An investigation by Pia et al. explores the association between ET-1, ET_A_R and ET_B_R in breast carcinoma [32]. Additionally, research in renal cell carcinoma (RCC) has also shown an increased expression in concentrations of ET-1 and ET_A_R [33]. Rosano et al. examined the role of ET-1 expression in ovarian cancer. The results from their research indicate an involvement of ET-1 and activation of MMP-2 and MMP-9 leading to the increased invasiveness of ovarian cancer cells [34]. Taken together, the data from these studies establish the significance of ET-1 in cancer.

HJP 272 is an ET receptor antagonist belonging to a series of 1,3,6-trisubstituted-4-oxo-1,4-hydroquioline-2-carboxylic acid analogs [35]. These analogs were synthesized and assessed for their selectivity towards ET_A_R. HJP 272 is the prototype of this series of molecules and, since its synthesis, its potential for the treatment of lung inflammation, preterm birth and microvascular hemorrhage in cerebral malaria has been demonstrated in preclinical models [[36], [37], [38], [39], [40]]. HJP 272, however, has not been previously investigated for its potential to treat cancer. Here, for the first time, we investigate its effects on two ET-1 and ET_A_R overexpressing cancer cell lines; A549 and MDA-MB-231.

## Methods

### Cell culture and chemicals

A549 non-small cell lung carcinoma (NSCLC) cells and MDA-MB-231 triple negative breast cancer (TNBC) cells were maintained in RPMI containing 10 % FBS and 1 % penicillin maintained at 37° C at 5 % CO2.

Ambrisentan was obtained from Fisher Scientific, Hampton, NH, USA. HJP 272 was synthesized using the previous protocol[35]. The structure of the final compound matched the previous nuclear magnetic resonance (NMR) spectroscopy.

### Cell viability assay

The anticancer effects of HJP 272 were determined by a 3-(4,5-dimethylthiazol-2-yl)-2,5-diphenyl-tetrazolium bromide (MTT) calorimetric assay. A549 and MDA-MB-231 cells were cultured and seeded into 96-well plates at a cell density of 2500 cells/well. Following 24 h of incubation for cell attachment, cells were treated with different concentrations of HJP 272 ranging from 0.75 µM to 100 µM. After 72 h of treatment, 100 µL of 5 mg/mL MTT solution were added to each well and the plates were then incubated at 37 °C for 2 h. The incubation was followed by aspirating the supernatants and adding 100 µL of DMSO to each well to dissolve the formazan crystals formed due to the reduction of MTT in the viable cells. The optical density (OD) was then measured at 570 nm using a BioTek™ Synergy H1 microplate reader (Winooski, VT, USA).

### NCI 60 analysis

HJP 272 was sent for NCI 60 screening (Bethesda, MD, USA). The compound was tested at a dose of 10^−5^ M and did not yield any significant growth inhibition in any of the 60 cancer cell lines (Supplementary Fig. 1).

### Transwell cell migration and invasion assay

The migratory capacity of MDA-MB-231 and A549 cells were assessed using the transwell chamber assay. Serum free media and cells (750,000 cells/well for MDA-MB-231 and 100,000 cells/well for A549) and the transwell inserts were placed in the upper chambers. FBS was added to the lower chambers and the wells were incubated for 24 h, followed by removal of media and gentle washing of the inserts with PBS (2X). The cells were then fixed with 4 % formaldehyde and permeabilized with 100 % methanol. Fixing was followed by staining with crystal violet (0.5 %). Non-migrated cells are scraped off gently with cotton swabs and then the migrated cells were imaged using an EVOS FL auto fluorescence microscope (Life Technologies Corporation, Carlsbad, CA, USA).

The invasiveness of A549 and MDA-MB-231 cells was investigated using transwells coated with matrigel and serum free media (1:40) and the same steps were followed to count the cells.

### Clonogenic assay

The *in vitro* colony formation capacity of the MDA-MB-231 cells was determined using a colony formation assay. Briefly, MDA-MB-231 cells were plated at a low density (250 cells/well) in 6-well plates and incubated overnight to allow for adherence. This overnight incubation was followed by treatment with HJP 272 or Ambrisentan at 50 or 100 µM. After 72 h of treatment, the culture media was replaced. The media was then replaced every two days for 10 days. On the 10th day, the colonies were washed twice with ice-cold PBS and fixed with 4 % formaldehyde for 15 min. Fixed cells were washed twice with ice-cold PBS and then stained with 0.5 % (w/v) crystal violet solution for 15 min. After the cells were stained, the colonies were washed with PBS, imaged, and counted, using colony counter CFU.

### Flow cytometric apoptosis assay

The apoptosis assay was performed using an NC0406119, Biolegend FITC Annexin V Apoptosis Detection Kit with 7-AAD and propidium iodide solution (PI). A549 and MDA-MB-231 cells were seeded, and apoptosis was induced with 100 µM HJP 272 for 48 h. The cells were collected and washed with 1x PBS and then centrifuged. The supernatant was carefully removed, and the cells were re-suspended in 1X binding buffer. Annexin V-FITC or PI solution was added to each population of cells. Positive control cells were incubated with both Annexin V-FITC and PI.

The percentages of apoptotic cells were quantified and extrapolated with the controls. Apoptosis was measured using each of the four quadrants and analyzed by using Flow Jo software Ver. 10 (BD Biosciences, Ashland, OR).

### EnzChek™ caspase 3/7 assay

Caspase 3/7 activity was measured using an EnzChek™ caspase-3 assay kit (Molecular Probes, Eugene, OR) and the assay was performed as per the manufacturer's specifications. Briefly, MDA-MB-231 and A549 cells were seeded at a density of 1 × 10^5^ cells per well in 24-well plates and treated with or without HJP 272 (100 µM) for 72 h. Cells were then harvested and the pellets were washed. The cells were lysed in 1X lysis buffer, incubated on ice for 30 min and centrifuged at 5000 rpm for 5 min. A 50 µl aliquot of the supernatant was then transferred to a 96 well plate to which 50 µL of 2x substrate working solution (10 mM of Z-DEVD-AMC substrate and 2x reaction buffer) was added. The 96-well plate was covered and incubated at 37 °C for 30 min. The fluorescence was then measured at 342/441 nm using a BioTek™ Synergy H1 microplate reader (Winooski, VT).

### RNA extraction by trizol reagent

Cell lines were treated with HJP 272 at a concentration of 100 µM for 72 h. Total RNA was isolated using the TRIzol reagent (Life Technologies, Inc). Cells were lysed with 0.5 mL of TRIzol reagent in 6-well plates and incubated on a shaker for 10 min with mild shaking. The incubation was followed by addition of chloroform and centrifugation at 16,000 g for 15 min at 4 °C. The aqueous phase was transferred to a fresh tube and 0.25 mL of isopropanol was added for RNA precipitation. The samples were then centrifuged at 16,000 g for 10 min at 4 °C. The RNA pellets were then washed with 75 % ethanol in DEPC water. The samples were mixed briefly and centrifuged at 12,000 g for 5 min at 4 °C. The supernatant was removed, and any traces of liquid were aspirated. The RNA pellets were allowed to air dry for 10 min and dissolved by adding 50 µL of DEPC water. The samples were then incubated at 4 °C overnight and the RNA concentration was measured using a picodrop spectrophotometer. Preparation of the RNA library and transcriptome sequencing were carried out by Novogene Co. LTD Beijing, China. A corrected P value < 0.05 was considered significant for each gene.

### Library construction, quality control and RNA sequencing

Messenger RNA was purified from total RNA using poly-T oligo-attached magnetic beads. After fragmentation, the first strand cDNA was synthesized using random hexamer primers, followed by the second strand cDNA synthesis using either dUTP for directional library or dTTP for non-directional library. The directional library was ready for use after end repair, A-tailing, adapter ligation, size selection, amplification, and purification; the non-directional library was ready for use after end repair, A-tailing, adapter ligation, size selection, USER enzyme digestion, amplification, and purification. The libraries were checked with Qubit and real-time PCR for quantification and with a bioanalyzer for size distribution detection. Quantified libraries were pooled and sequenced on Illumina platforms. The clustering of the index-coded samples was performed according to the manufacturer’s instructions. After cluster generation, the library preparations were sequenced on an Illumina platform and paired-end reads were generated.

### RNA sequencing data analysis

Raw data (raw reads) of fastq format were first processed through fastp software. In this step, clean data (clean reads) were obtained by removing reads containing adapter, reads containing ploy-N and low-quality reads from raw data. At the same time, Q20, Q30 and GC content of the clean data were calculated. All the downstream analyses were based on high-quality clean data.

Reference genome and gene model annotation files were downloaded from the genome website directly. The index of the reference genome was built using Hisat2 v2.0.5 and paired-end clean reads were aligned to the reference genome using Hisat2 v2.0.5. Hisat2 was selected as the mapping tool because it can generate a database of splice junctions based on the gene model annotation file and thus produces a better mapping result than other non-splice mapping tools.

FeatureCounts v1.5.0-p3 was used to count the reads mapped to each gene. Fragments Per Kilobase of transcript sequence per Million base pairs sequenced (FPKM) of each gene was calculated based on the length of the gene and the reads count mapped to the gene. FPKM considers the effect of sequencing depth and gene length for the reads count at the same time and is currently the most used method for estimating gene expression levels.

Differential expression analysis of two conditions/groups (two biological replicates per condition) was performed using the DESeq2 R package (1.20.0). DESeq2 provides statistical routines for determining differential expression in digital gene expression data using a model based on the negative binomial distribution. The resulting P-values were adjusted using the Benjamini and Hochberg’s approach for controlling the false discovery rate. Genes with an adjusted P-value <0.05 found by DESeq2 were assigned as differentially expressed.

For edgeR without biological replicates, prior to differential gene expression analysis, for each sequenced library, the read counts were adjusted by an edgeR program package through one scaling normalized factor. Differential expression analysis of two conditions was performed using the edgeR R package (3.22.5). The P values were adjusted using the Benjamini & Hochberg method. Corrected P-values of 0.05 and absolute foldchanges of 2 were set as the threshold for significantly differential expression.

Gene Ontology (GO) enrichment analysis of differentially expressed genes was implemented by the clusterProfiler R package, in which gene length bias was corrected. GO terms with corrected P values <0.05 were considered significantly enriched by differentially expressed genes. Kyoto Encyclopedia of Genes and Genomes (KEGG) is a database resource for understanding high-level functions and utilities of biological systems, such as cells, organisms and ecosystems, from molecular-level information, especially large-scale molecular datasets generated by genome sequencing and other high-throughput experimental technologies (http://www.genome.jp/kegg/). A clusterProfiler R package was used to test the statistical enrichment of differentially expressed genes in KEGG pathways. The Reactome database brings together the various reactions and biological pathways of human model species. Reactome pathways with corrected P values <0.05 were considered significantly enriched by differentially expressed genes. The DO (Disease Ontology) database describes the function of human genes and diseases. DO pathways with corrected P values <0.05 were considered significantly enriched by differentially expressed genes. The DisGeNET database integrates human disease-related genes. DisGeNET pathways with corrected P values <0.05 were considered significantly enriched by differentially expressed genes. ClusterProfiler software was used to test the statistical enrichment of differentially expressed genes in the Reactome pathway, the DO pathway, and the DisGeNET pathway.

Gene Set Enrichment Analysis (GSEA) is a computational approach to determine if a pre-defined Gene Set can show a significant consistent difference between two biological states. The genes were ranked according to the degree of differential expression in the two samples, and then the predefined Gene Sets were tested to see if they were enriched at the top or bottom of the lists. Gene set enrichment analysis can include subtle expression changes. The local version of the GSEA analysis tool (http://www.broadinstitute.org/gsea/index.jsp) was used on the GO, KEGG, Reactome, DO and DisGeNET datasets.

GATK (v4.1.1.0) software was used to perform single nucleotide polymorphism (SNP) calling. Raw vcf files were filtered with the GATK standard filter method and other parameters (cluster: 3; WindowSize :35; QD < 2.0; FS > 30.0; DP < 10. rMATS(4.1.0) software was used to analyze alternative splicing events. Protein-Protein Interaction (PPI) analysis of derived from differentially expressed genes was based on the STRING database, with known and predicted PPIs.

A fusion gene is a chimeric gene formed by the fusion of all or part of the sequences of two genes, generally caused by chromosome translocation, deletion or other reasons. STARfusion software (1.9.0) was used to detect genes that are fused. STARfusion is a software package that uses fusion output results of STAR alignment to detect fusion transcripts, including STAR alignment. STAR-fusion.filter was used to correct the predicted results of STARfusion to ensure the accuracy of the results. The RNAseq data have been deposited in the Mendeley data repository (https://data.mendeley.com/preview/vfkx4pj2dm?a=8e344e13-97f0-4fb7-bd55-eb7336677c62).

### Western blot assay

A549 and MDA-MB-231 cells were treated with HJP 272 (50 or 100 µM) or Ambrisentan (50 or 100 µM) for 72 h. After treatment, the cells were trypsinized, washed with PBS and centrifuged at 5000 rpm for 5 min. The cells were then lysed with RIPA buffer and 1x Halt™ Protease and Phosphatase Inhibitor Cocktail and incubated on ice for 30 min. After incubation on ice, the cells were centrifuged at 12,000 rpm for 20 min at 4 °C. The supernatant was then collected, and cell lysate protein content was determined using a BCA assay. Equal concentrations of cell lysate protein (20 µg) were resolved using 4–20 % gradient gels (Bio-Rad, Hercules, CA, USA) and transferred to PVDF membranes that were soaked in 100 % methanol. The membranes were then blocked with 5 % nonfat dry milk prepared in TBST (10 mM Tris–HCl, pH 8.0, 150 mM NaCl and 0.1 % Tween 20) for 1 h at room temperature and then left overnight at 4 °C on a shaker with primary monoclonal antibodies. The membranes were washed with TBST and incubated with horseradish peroxide (HRP)-conjugated secondary antibody in 5 % nonfat dry milk on a shaker at room temperature for 1 hr. The bands were visualized using SuperSignal™ West Femto Maximum sensitivity substrate (Azure Biosystems, Dublin, CA, USA and LI-COR Biosciences, Lincoln, NE, USA) and analyzed using ImageJ software (Bethesda, MD, USA). Ambrisentan, a United States Food and Drug Administration (FDA) approved, ET_A_R antagonist, was used as a positive control. The antibodies used were glyceraldehyde-3-phosphate dehydrogenase (GAPDH, Cell Signaling, Beverly, MA, USA # 2118S), endothelin-converting enzyme-1 (ECE-1, Santa Cruz, CA # sc-376018), p38 mitogen-activated protein kinase (p38 MAPK, Cell Signaling #9212S), CDK6 (Santa Cruz # sc-53638), α/β-Tubulin (Cell Signaling #2148S), IL-6 (Cell Signaling # 12153S), Anti-mouse IgG, HRP-linked antibody (Cell Signaling #7076S), Anti-rabbit IgG, HRP-linked antibody (Cell Signaling #7074S).

### 3D-spheroid assay

A549 and MDA-MB-231cells were seeded in 96-well, Nunclon Sphera-Treated, U-Shaped-Bottom Microplates, Nunclon™ Sphera™ Cat # 174925, Thermo Scientific, Waltham, MA, USA at 1500 cells/well, centrifuged at 1000 rpm for 5 min and incubated at 37 °C overnight to achieve 3D spheroids. The spheroids were then treated every 72 h with either HJP 272 or Ambrisentan at 50 or 100 µM. The spheroids were imaged every 3 days using a Nikon Eclipse Ts2R-FL camera, (Melville, NY, USA).

The experiment involving 3D spheroid assay with MDA-MB-231 is a result of two successful trials. The assay was conducted at *n* = 4. Beyond day 9, the spheroids grew to a size where they became fragile leading to fragmentation of the cell aggregates. We were able to detect a trend in spheroid size that closely matched the outcomes from the previous trials. However, a precise measurement of the spheroids was not attainable and therefore the graphs represented in Fig. 7 show the values of the two trials (*n* = 2).

### Live-dead cell assay

On day 21, following the imaging of the spheroids, cell viability was measured using a viability/cytotoxicity assay kit for live and dead animal cells (Cat #30002-T, Biotium, Fremont, CA, USA). The kit components include Calcein AM, (4 mM in DMSO) and EthD-III, (2 mM in DMSO/H_2_O). Based on the manufacturer's instructions, a solution using the dyes was made in PBS and added to the 96-well plates containing the spheroids, which were then imaged using a Nikon Eclipse Ts2R-FL camera. Calcein AM in live cells emits a green fluorescence, while EthD-III penetrates dead cells, staining the nucleus with a red fluorescence. The stained spheroids were merged and imaged.

### Statistical analysis

All experiments were performed at least two times. The statistical significance between two groups was determined with Student’s *t*-test, whereas comparisons among multiple groups was carried out by one-way ANOVA. *P* < 0.05 was considered statistically significant.

## Results

### Cytotoxic effect of HJP 272 in A549 and MDA-MB-231 cells

The cytotoxic effect of HJP 272 in A549 and MDA-MB-231 cells is represented in Supplementary Fig. 2**.** The graph was generated using GraphPad Prism 9 by a non-linear regression (curve-fit) model. Treatment with up to 100 μM of HJP 272 had no effect on the viability of either type of cancer cell.

### HJP 272 causes significant inhibition of migration and invasion in A549 and MDA-MB-231 cells

**Fig. 1:**
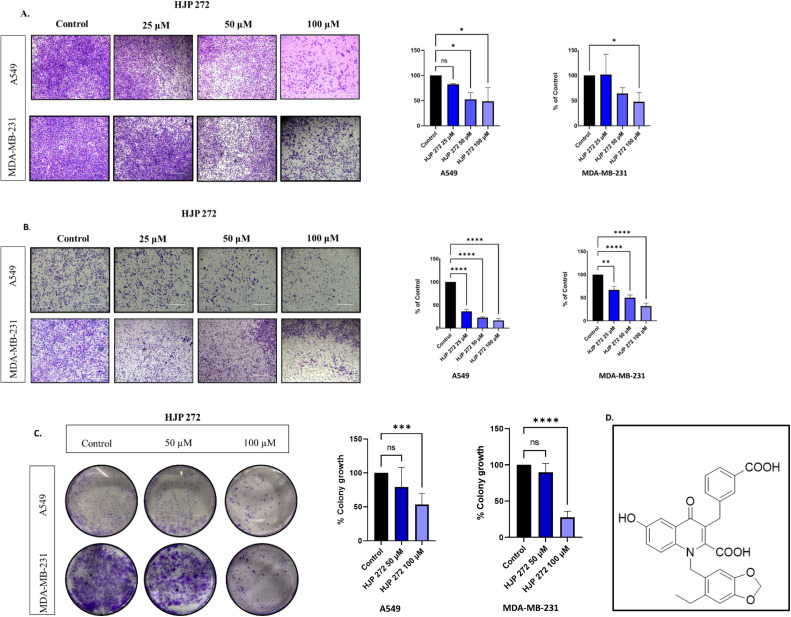
**HJP 272 prevents migration, invasion and colony formation of A549 and MDA-MB-231 cells.** (A) Boyden chamber assay showing the anti-migratory effects of HJP 272 in A549 and MDA-MB-231 cells and their graphical representations. (B) Boyden Chamber assay showing the anti-invasive effects of HJP 272 in A549 and MDA-MB-231 cells and their graphical representations. (C) Clonogenic assay in A549 and MDA-MB-231 treated cells. *p < 0.05, **p < 0.01, ***p < 0.001. (D) Chemical structure of HJP-272.

A Boyden chamber assay was performed to analyze the effects of HJP 272 on migration and invasion in both cell lines. Treatment of A549 and MDA-MB-231 cell lines with HJP 272 at concentrations of 25 µM, 50 µM or 100 µM for 24 h showed a significant decrease in the percentage of cells that migrated or invaded the transmembrane in a concentration dependent manner (Fig. 1A and B).

### HJP 272 causes significant reduction in colony formation capacities of A549 and MDA-MB-231 cells

The effect of HJP 272 on colony formation was tested with clonogenic assays. After 72 h, colonies were allowed to grow for 10 days, and the percentage of colonies that survived after treatment with 50 or 100 µM was recorded. Representative images show a significantly smaller number of colonies after treatment with HJP 272 (Fig. 1C). At 100 µM, HJP 272 reduced colony formation by ∼1.61- and ∼3.53-fold in A549 and MDA-MB-231 cells, respectively. The chemical structure of HJP 272 is shown in Fig. 1D

### HJP 272 induces apoptosis in A549 and MDA-MB-231 cells

**Fig. 2:**
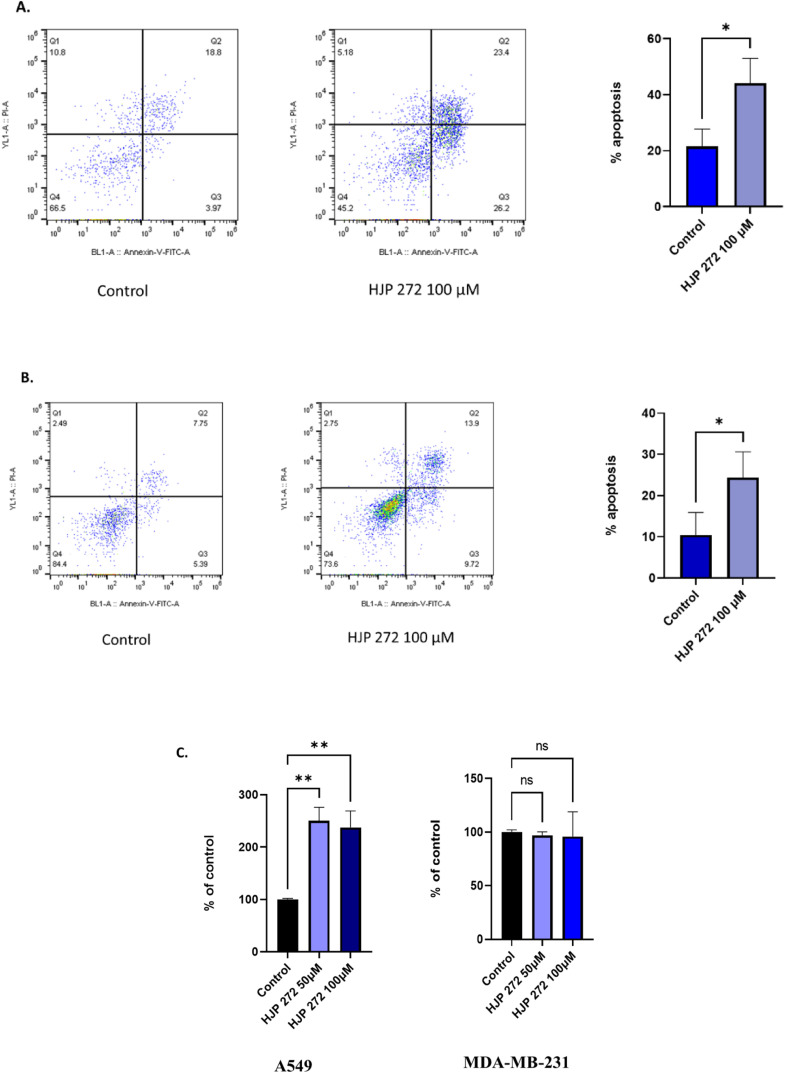
**HJP 272 increases apoptosis in A549 and MDA-MB-231 cells.** Apoptosis assay showing the effect of 48-hour treatment with 100 µM HJP 272 in (A) A549 and (B) MDA-MB-231 cells. Total percentage of apoptotic cells after treatment with HJP 272 compared to controls. The figures are representative of three independent experiments. (C) EnzChek™ Caspase-3 activity assay showing the levels of caspase-3 in comparison to the control. Statistical significance was defined as *p < 0.05, **p < 0.01.

To further investigate the role of HJP 272 in cancer, an apoptosis assay using flow cytometry was performed in A549 and MDA-MB-231 cells. Cells were treated with HJP272 at 100 µM for 48 h. The percentage of early and late apoptosis was measured and was found to be significantly higher in the treatment group compared to the control in both cell lines (Fig. 2A and B). Percentages of apoptotic cells (late and early) were 44.13 % and 24.39 % in A549 and MDA-MB-231 cells respectively, compared to their respective controls, which were 21.46 % and 10.4 %.

### EnzChek™ assay to determine caspase-3 activity

The effect of HJP 272 on caspase-3 activity in A549 and MDA-MB-231 was tested with an EnzChek™ caspase-3 assay kit. A549 cells treated with HJP 272 at 50 or 100 µM for 48 h showed ∼1.5- and ∼1.4-fold increases in fluorescence intensity compared to control cells, while no significant changes were observed in MDA-MB-231 cells (Fig. 2C).

### Effect HJP 272 on A540 and MDA-MB-231 transcriptomes

**Fig. 3:**
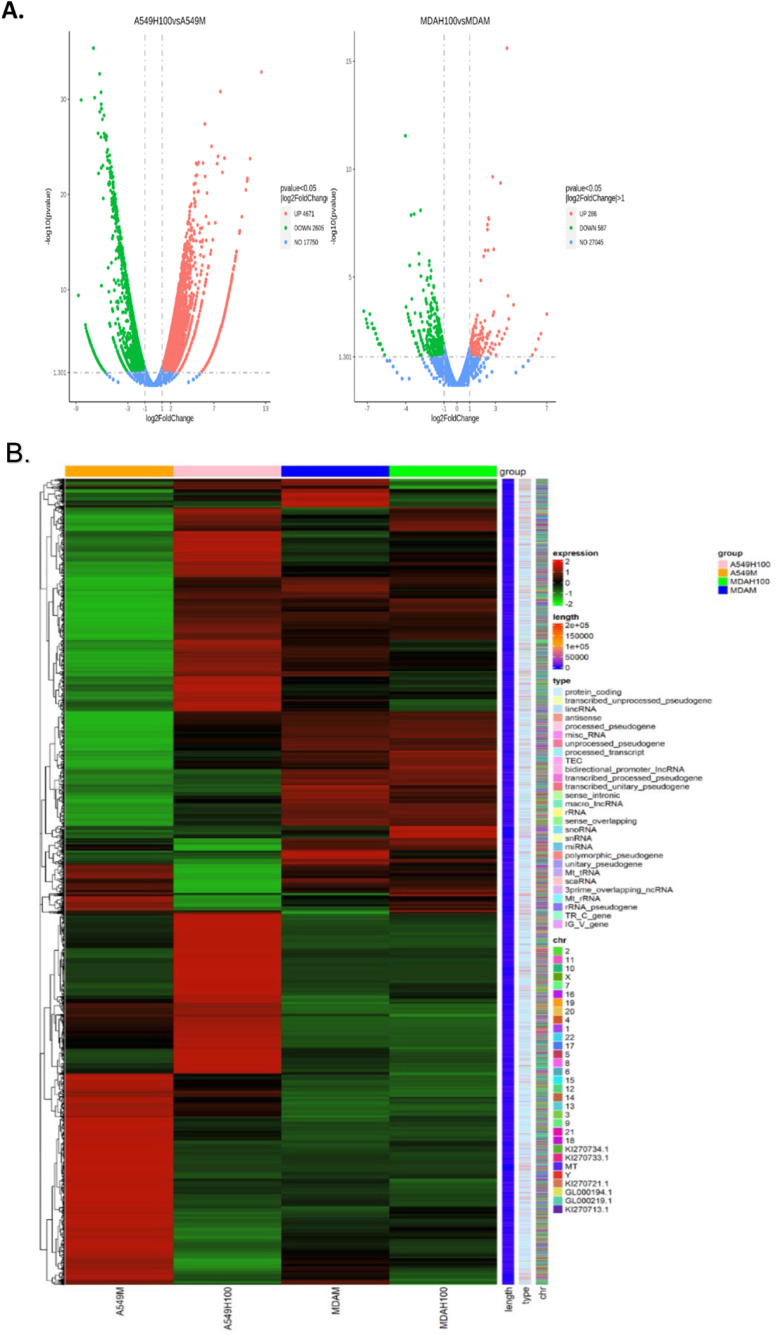
**HJP 272 affects expression of genes involved in cell proliferation and metastasis in A549 and MDA-MB-231 cells.** (A) Volcano plots for A549 (left) and MDA-MB-231 (right) cells. The abscissa in the figure is log2 fold-change, and the ordinate is -log10 padj or -log10 *p* value. The blue dashed line indicates the threshold for differential expression. (B) Heatmap of hierarchical clustering shows the transcriptome profiles of A549 and MDA-MB-231 cells in the absence or presence of HJP 272 at 100 µM. The overall results of FPKM cluster analysis, clustered using the log2(FPKM+1) value. Red color indicates genes with high expression levels, and green color indicates genes with low expression levels. The color ranging from red to green indicates the log2(FPKM+1) values from large to small. The chromosome to which each gene belongs, gene's length, and the biological type of the gene are also added to the heatmap.

**Fig. 4:**
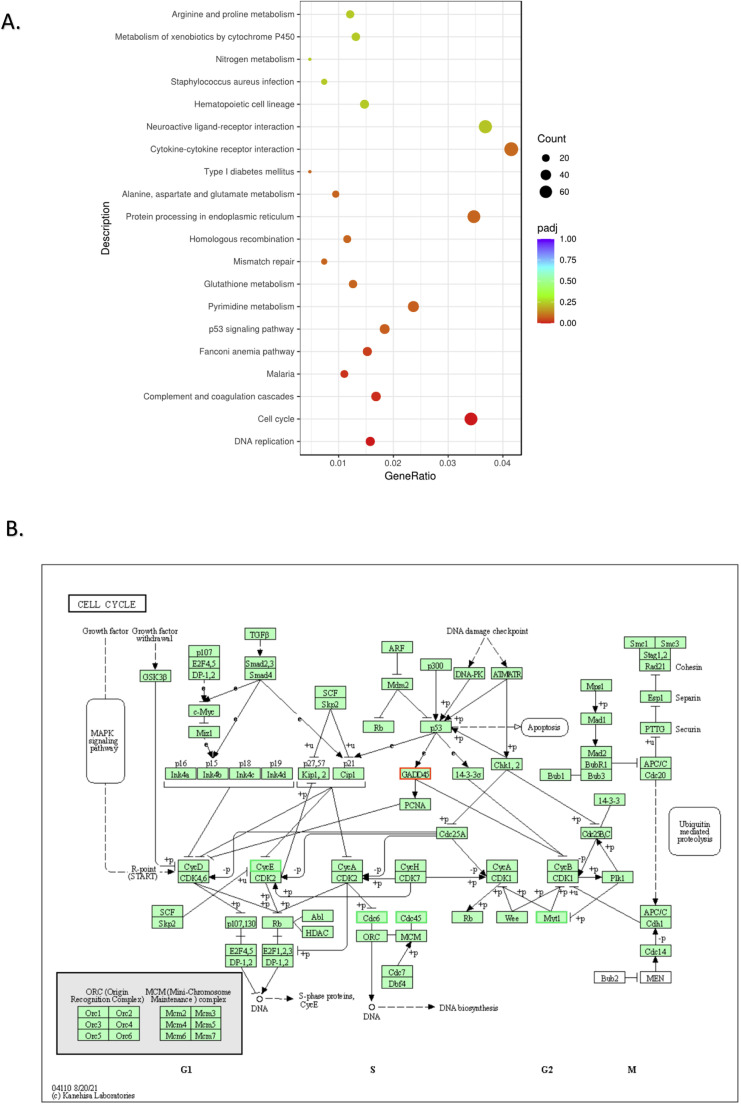
**KEGG pathway analysis of the effect of HJP 272 on A549 cells.** (A) KEGG enrichment scatter plot. The abscissa in the graph is the ratio of the number of differential genes on the KEGG pathway to the total number of differential genes and the ordinate is KEGG pathway. (B) Cell cycle pathway map. The downregulated genes (CycE, Cdc6 and Cdc 45) are in green rectangles and the up-regulated gene (GADD45) is in a red rectangle.

**Fig. 5:**
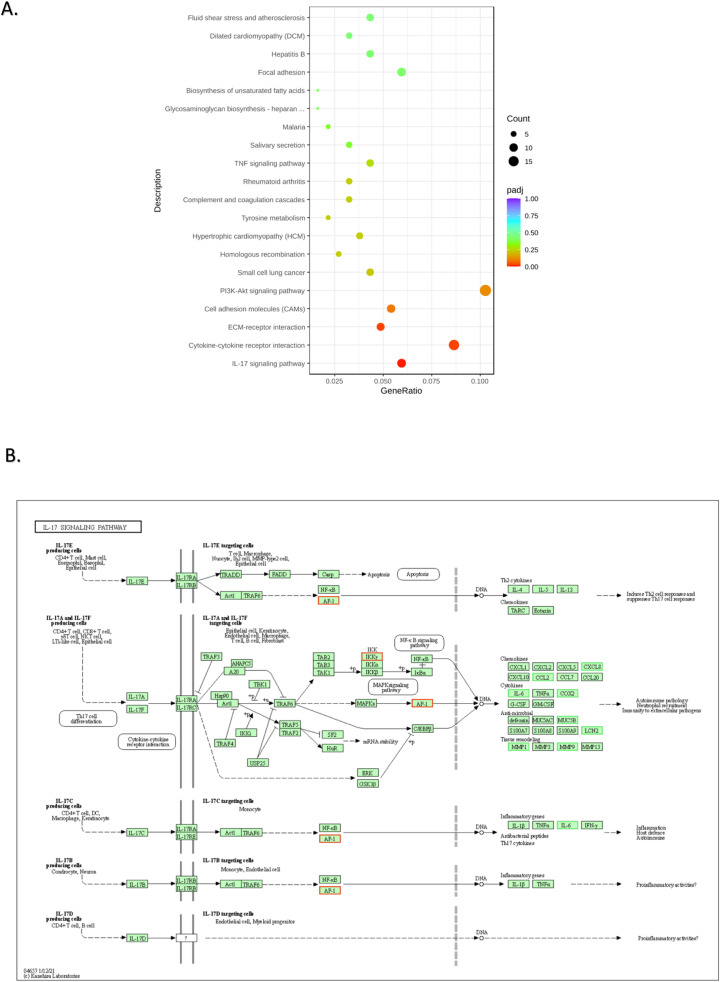
**KEGG pathway analysis of the effect of HJP 272 on MDA-MB-231 cells.** (A) KEGG enrichment scatter plot. The abscissa in the graph is the ratio of the number of differential genes on the KEGG pathway to the total number of differential genes and the ordinate is KEGG pathway. (B) IL-17 signaling pathway map. The downregulated genes (CCL2, IL-6, COX2, MMP1 and MMP9) are in green rectangles and the up-regulated gene (AP-1 and IKKγ) is in a red rectangle.

Next, we evaluated the effects of blocking the ET_A_R with HJP 272 at 100 µM in A549 and MDA-MB-231 cells by RNA sequencing (RNA-seq). RNA was isolated and checked for quality using picodrop. RNA was further evaluated for yield, purity and integrity and a cDNA library was constructed by Novogene. Volcano plots (Fig. 3A) indicate the numbers of genes significantly upregulated or downregulated after treatment with HJP 272 in A549 and MDA-MB-231 cells. Differences in gene expression between HJP 272-treated and untreated cells were further analyzed using hierarchical clustering (Fig. 3B). As seen in Fig. 3, HJP 272 produced major effects in gene expression in A549 cells. Pathway analysis revealed downregulation of cancer cell proliferation and metastatic pathways. Specifically, HJP 272 decreased expression of CDK-Rb-E2F axis genes. KEGG enrichment pathway analysis revealed that the two most significantly downregulated pathways in A549 cells were DNA replication and cell cycle pathways (Fig. 4A), consistent with HJP 272′s inhibitory effect on metastasis. The KEGG generated cell cycle pathway map reveals that HJP 272 significantly down-regulated cyclin E (CycE), a critical cell cycle gene which facilitates the transition from G1 to M phase, as well as two important cell division cell cycle genes (Cdc6 and Cdc45) (Fig. 4B). Importantly, HJP 272 up-regulated the gene that encodes growth arrest and DNA damage-inducible protein 45 (GADD45), a protein which plays a crucial role in DNA repair, cell cycle regulation and apoptosis. While effects on gene transcription in MDA-MB-231 were less significant, HJP 272 decreased expression of IL-17 signaling pathway genes IL-6, COX2 and MMP9. HJP 272 also downregulated members of the CXC gene subfamily and genes encoding extracellular matrix proteins, including collagen and fibronectin in this cell line. In MDA-MB-231 cells, KEGG enrichment pathway analysis indicated that the IL-17 signaling pathway was the most down-regulated pathway by HJP-272 treatment (Fig. 5A). The IL-17 signaling pathway map reveals down-regulation of IL-6 and CCL2 and, relevant to HJP 272′s anti-metastatic function, the extracellular matrix degrading enzymes MMP1 and MMP9 (Fig. 5B). The effect of HJP 272 on key pathways is further explored in both cell lines by testing for changes at the protein level.

### Potential mechanism of action of HJP 272

**Fig. 6:**
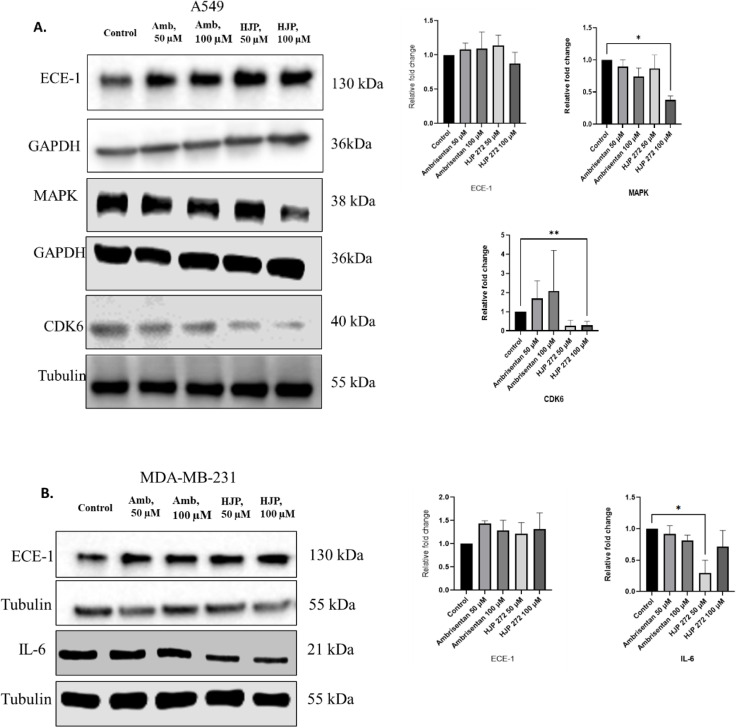
**HJP 272 decreases levels of key mediators of cancer progression.** (A) Western blots in A549 cells to detect the expression of ECE-1, CDK6 and ECE-1. (B) Western blots in MDA-MB-231 cells to detect the expression of ECE-1 and IL-6. The graphs represent the mean ± *S*.D. of the respective protein bands as a ratio of protein of interest to α/β-tubulin or GAPDH. *p < 0.05, **p < 0.01 compared to the control. Amb, ambrisenten; HJP, HJP 272; ECE-1, endothelin-converting enzyme-1; MAPK, mitogen-activating protein kinase; CDK6, cyclin dependent kinase 6; IL-6, Interleukin-6.

Treatment with 100 µM HJP 272 in both cell lines resulted in downregulation of specific molecular pathways and genes implicated in cell cycle regulation, extracellular matrix degradation and metastasis as shown in the RNA-seq analysis described above. Western blot analysis showed downregulation of CDK6 and MAPK in A549 cells (Fig. 6A), while IL-6 was significantly decreased in MDA-MB-231 cells treated with 50 μM HJP 272. (Fig. 6B). HJP 272 did not affect levels of ECE-1 in either cell line (Fig. 6A and B).

### HJP272 inhibits spheroid tumor growth in MDA-MBA-231 cells

**Fig. 7:**
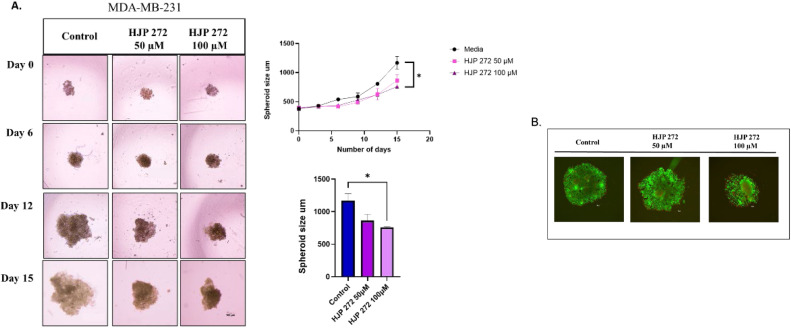
**HJP 272 reduces MDA-MD-231 spheroid growth.** (A) MDA-MB-231cells were seeded in 96-well, Nunclon Sphera-Treated, U-Shaped-Bottom Microplates at 1500 cells/well, centrifuged at 1000 rpm for 5 min and incubated at 37 °C overnight to achieve 3D spheroids. The test spheroids were then treated every 72 h with either HJP 272 at 50 or 100 µM. The spheroids were imaged every 3 days using a Nikon Eclipse Ts2R-FL camera. Graphs show mean + SEM of sizes of spheroids treated or untreated with HJP 272 over 15 days (top) and at the end of the experiment (bottom). The graphs are representative of two independent experiments. (B) A Biotium viability/cell toxicity kit was used to distinguish live from dead cells. Calcein AM (green) indicates live cells; EthD-III indicates dead cells. *p < 0.05.

Treatment with HJP272 at 100 µM decreased spheroid diameter by 35.15 % compared to controls on day 15 in MDA-MB-231 cells (*p* < 0.05, Fig. 7A). On the other hand, A549 did not affect spheroid size for up to 9 days (Supplementary Fig. 3). Spheroids were imaged towards the end of the experiment on day 21 using the two-color assay to measure cell viability and distinguish between the live and dead cells in the treated spheroids. The representative images in Fig. 7B show MDA-MB-231 cells with mostly green fluorescence (live cells) in the control spheroids, while some red fluorescence (dead cells) can be observed in the HJP 272 treated cells at 50 and 100 µM.

## Discussion

The role of ET-1 and its receptors in cancer pharmacology is becoming increasingly evident [41]. ET-1 is overexpressed in a wide range of cancers and the ET-axis has emerged as a potential therapeutic target. Some of the strategies to target the ET-axis in cancer include ECE inhibition and selective and non-selective antagonism of the two ET receptor subtypes, ET_A,_ and ET_B_ [42]. The present study focuses on testing HJP 272, an ET_A_ receptor antagonist which was originally synthesized by our group [35], for its ability to decrease cancer proliferation and migration.

We hypothesized that HJP 272 would mediate its anti-cancer effects at the level of gene transcription. In fact, RNA-seq of A549 cells treated with HJP 272 displayed downregulation of pathways involved in cancer cell proliferation and metastasis. Specifically, HJP 272 at 100 µM reduced expression of the CDK-Rb-E2F axis. CDK inhibits tumor suppressor retinoblastoma (Rb), leading to activation of transcription factor E2F and progression of the cell cycle [43]. E2F is normally bound to Rb, but once Rb is phosphorylated by CDK, it releases E2F, enabling the transcription factor to promote expression of a range of genes involved in cell proliferation [44]. CDK6 is a member of the CDK family that binds to cyclin D and is reported to be involved in proliferation and apoptosis of NSCLC cells [45]. Taken together, the western blot and RNA-seq data show downregulation of CDK6 in HJP 272 treated A549 cells, indicating a potential mechanism whereby this compound inhibits cancer cell progression and apoptosis. Additionally, mitogen-activated protein kinases (MAPKs) have a conspicuous role in cellular processes related to cancer progression, such as proliferation, cell death, migration, and invasion [46]. Although RNA-seq results did not show any significant downregulation of MAPK in HJP 272 treated A549 cells, western blot analysis revealed a significant downregulation in MAPK protein expression. Finally, the two most down-regulated gene pathways in A549 cells treated with HJP 272 were the DNA replication and cell cycle pathways, which are critical for cancer cell metastasis. Moreover, HJP 272 decreased the expression of cell cycle genes that drive cell division, *i.e.*, CycE, Cdc6 and Cdc45, and increased expression of the pro-apoptotic gene GADD45.

Similarly, ET_A_R blockade in MDA-MB-231 cells with HJP 272 treatment revealed several important changes in the transcriptome profile of these cells, such as downregulation of IL-6, COX2 and MMP9, components of the IL-17 signaling pathway. Further, HJP 272 reduced the expression of genes in the CXC subfamily and genes encoding fibronectin and collagen, components of the ECM, which impede cancer cell invasion and metastasis. Interestingly, HJP 272 did not affect the expression of ECE-1 in either cell line, indicating that our compound does not regulate ET-1 through ECE-1.

CXC chemokine ligands and receptors are involved in regulating cancer angiogenesis and metastasis. This finding is consistent with our group’s earlier study where pretreatment with HJP 272 significantly reduced CXCL12 levels in bleomycin (Bleomycin)-induced pulmonary fibrosis animal models [38]. Additionally, downregulation of IL-6 through the IL-17 signaling pathway would be expected to decrease cell proliferation in breast cancer [47]. The RNA-seq data showing downregulation of ECM components and the potential for inhibition of cancer invasion is supported by the Boyden chamber assay where treatments with HJP 272 in MDA-MB-231 at concentrations of 50 and 100 µM showed significant reductions in the migration and invasion capacities of these cells after 24 h of treatment. The RNA-seq results showing down-regulation of DNA replication and cell cycle pathways as well as the IL-17 signaling pathway provides a mechanistic explanation for HJP 272′s effects on proliferation, migration and invasion shown in the accompanying experiments presented here. HJP-272′s effect on key metastasis driving genes provides further support for its role in preventing migration and invasion of cancer cells by altering the transcriptomes of those cells via its ET-1 blocking effect.

We also evaluated the effect of HJP 272 on cancer cell colony formation. Clonogenic assay assesses the ability of a single cell to grow into a colony, testing the ability of every cell in the population to divide and essentially quantifies reproductive cell survival *in vitro* [48,49]. Clonogenic assay helps measure a cell’s reproductive capacity over a prolonged period, providing the vital phenotypic characteristics to develop following multiple cell divisions. HJP-272 decreased the colony forming capacity of both A549 and MDA-MB-231 cells at 100 µM.

Protection from apoptosis can be a contributing factor to tumor growth and progression [50], evidenced by the findings of Donatella et al. in ovarian carcinoma cells [51]. Additionally, research conducted by Nelson et al. (2005), revealed that cell survival in prostate cancer could be a consequence of ET-1 signaling through the Erk1/2 pathway [52]. These investigators also showed that treatment of prostate cancer cells with ET-1 caused a downregulation of some of the apoptotic proteins such as Bad, Bax and Bak, suggesting the correlation of increased expression of ET-1 and cell survival in cancer cells [52]. Therefore, we hypothesized that blocking ET_A_ receptors could be proapoptotic and evaluated the apoptotic capability of HJP 272 through flow cytometry and EznCheck™ assays. A significant number of cells underwent apoptosis compared to the control after 48 h of treatment with HJP 272 at 100 µM in both A549 and MDA-MB-231 cell lines. Additionally, caspase-3-mediated apoptosis activity was found to be increased in A549 cells treated with 50 or 100 µM HJP-272 for 72 h, although no significant change was observed in MDA-MB-231 cells at the same concentrations and time point. Therefore, the apoptotic activity seen in A549 through flow cytometry could be attributed to an increase in caspase 3 activity.

The 3D spheroid assay helps generate *in vitro* models that are clinically and biologically relevant[53]. HJP 272 at 100 μM reduced tumor size in MDA-MB-231 cells after 15 days, while A549 cells did not show any significant change following treatment for 9 days. Cells within the spheroids are attached to each other through secreted ECM, which assists in the formation of a complex structure that closely resembles an *in vivo* tissue system [54]. Collagen and fibronectin are the two major proteins of the ECM, providing structural integrity and regulating several cellular processes such as growth, migration, morphogenesis etc. [55]. Interestingly, treatment of MDA-MB-231 cells with HJP 272, but not A549 cells, demonstrated decreased expression of genes encoding both collagen and fibronectin. These results that are in line with the changes observed in the 3D spheroid assay in MDA-MB-231 cells, but not A549 cells.

The use of only two cell lines in this work is a potential weakness of the study. Testing more cell lines would increase the power and generalizability of the data. However, this is the first paper exploring the anti-metastatic potential of HJP 272 and the data here have set the stage for future investigation with additional cell lines. Also, cell lines that are very distinct from each other were selected intentionally. Both cell lines are well characterized, have aggressive phenotypes and represent clinically challenging cancer types. A549 cells, a non-small cell lung cancer cell line, is derived from an epithelial adenocarcinoma of the lung, a type of lung cancer that accounts for most lung cancers. These cells are epithelial. MDA-MB-231 cells are triple negative breast cancer cells, which are notoriously metastatic and difficult to treat. These cells are mesenchymal. We selected diverse cell lines (one epithelial line, one mesenchymal line; one lung cancer line, one breast cancer line), as we are testing for broad anti-metastatic potential across diverse types of cancer.

Another potential weakness of this report is the lack of *in vivo* studies. Of course, *in vitro* cancer models do not consider how drug efficacy may be affected when the host’s homeostasis has been hijacked by the cancer [56]. However, in this initial study, we have deliberately focused on detailed investigations to elucidate the cellular and molecular mechanisms of HJP 272′s effect on cancer cell metastasis. In fact, the complexity and variability inherent in metastatic *in vivo* models can often interfere with understanding the molecular mechanism of action of a new or re-purposed compound. By focusing first on clearly defined cellular models, we have ensured a rigorous and controlled analysis of drug action, which will be essential for interpreting outcomes in subsequent *in vivo* studies.

## Conclusion

The role of ET-1 in various pathological conditions has been established over the years. HJP 272, an ET_A_ receptor antagonist previously synthesized by our group, has been shown to have favorable effects in preclinical *in vivo* models of pulmonary fibrosis, preterm birth and cerebral malaria. We hypothesized that ET-1 plays a role in various molecular pathways involved in cancer progression and we therefore decided to investigate the potential role of HJP 272 in preventing cancer metastasis and invasion. Several ET receptor antagonists, such as Ambrisentan and Macitentan, have been shown to inhibit cancer cell migration and invasion. HJP 272, a prototype analog that is a noncompetitive antagonist with the capability of binding to ET_A_ [40], is an addition to this line of ERAs [[57], [58], [59]]. While ERAs show anti-neoplastic activity *in vitro*, for the most part, clinical trials have not reflected those findings [58].

Although the current study demonstrates the effect of HJP 272 on cancer cell migration, invasion, and proliferation *in vitro*, cell migration is a complex biological process, driven by interactions of cells with ECM and other neighboring cells. Therefore, live-cell imaging [57] and *in vivo* studies are needed to test HJP 2762′s clinical potential. Finally, the possibility of synergism through the use of combination therapy for cancer which includes HJP 272 should be explored.

## Data availability

Raw data underlying this article are available from the corresponding author upon reasonable request.

## Funding

This work was supported by a funds awarded to SER by Nassau University Medical Center, Nassau, NY. SER is supported by a grant from the NIH: 1R16GM145586.

## Acknowledgements

We are very grateful to Dr. Zhe-Sheng Chen for providing the cell lines. This work is dedicated to the memory of Dr. Ralph A. Stephani and Dr. Hardik J. Patel, who originally synthesized HJP 272.

## CRediT authorship contribution statement

**Nabeela Baig:** Data curation, Formal analysis, Investigation, Methodology, Project administration, Validation, Visualization, Writing – original draft. **Rameswari Chilamakuri:** Methodology. **Saurabh Agarwal:** Methodology. **Aaron Muth:** Conceptualization, Data curation, Formal analysis, Investigation, Methodology, Project administration, Resources, Supervision, Validation, Visualization. **Sandra E. Reznik:** Conceptualization, Data curation, Formal analysis, Funding acquisition, Methodology, Project administration, Resources, Supervision, Validation, Visualization, Writing – review & editing.

## Declaration of competing interest

The authors declare that they have no known competing financial interests or personal relationships that could have appeared to influence the work reported in this paper.

## Appendix Supplementary materials

Image, application 1

Image, application 2

Image, application 3

## References

[1] Shihoya W., Nishizawa T., Okuta A., Tani K., Dohmae N., Fujiyoshi Y. (2016). Activation mechanism of endothelin ET B receptor by endothelin-1. Nature.

[2] Wang Z., Liu P., Zhou X., Wang T., Feng X., Sun Y.P. (2017). Endothelin promotes colorectal tumorigenesis by activating YAP/TAZ. Cancer Res..

[3] Benigni A., Buelli S., Kohan D.E. (2021). Endothelin-targeted new treatments for proteinuric and inflammatory glomerular diseases: focus on the added value to anti-renin-angiotensin system inhibition. Pediatr. Nephrol..

[4] Stow L.R., Jacobs M.E., Wingo C.S., Cain B.D. (2011). Endothelin-1 gene regulation. FASEB J..

[5] Opitz C.F., Ewert R., Kirch W., Pittrow D. (2008 Aug). Inhibition of endothelin receptors in the treatment of pulmonary arterial hypertension: does selectivity matter?. Eur. Heart. J..

[6] Davenport A.P., Hyndman K.A., Dhaun N., Southan C., Kohan D.E., Pollock J.S. (2016). Endothelin. Pharmacol. Rev..

[7] Houde M., Desbiens L., D’Orléans-Juste P. (2016). Endothelin-1: biosynthesis, signaling and vasoreactivity. Adv. Pharmacol..

[8] Kawanabe Y., Nauli S.M. (2011). Endothelin. Cell. Molec. Life Sci..

[9] Kedzierski R.M., Yanagisawa M. Endothelin System: the double-edged sword in health and disease [Internet]. 2001. Available from: www.annualreviews.org. 10.1146/annurev.pharmtox.41.1.851 11264479

[10] Wu M.H., Lo J.F., Kuo C.H., Lin J.A., Lin Y.M., Chen L.M. (2012). Endothelin-1 promotes MMP-13 production and migration in human chondrosarcoma cells through FAK/PI3K/akt/mTOR pathways. J. Cell Physiol..

[11] Masaki T. (2004). Historical review: endothelin. Trends Pharm. Sci..

[12] Braasch I., Schartl M. (2014). Evolution of endothelin receptors in vertebrates. Gen. Comp. Endocrinol..

[13] Slottosch I., Liakopoulos O., Kuhn E., Deppe A., Lopez-Pastorini A., Schwarz D. (2014). Controlled lung reperfusion to reduce pulmonary ischaemia/reperfusion injury after cardiopulmonary bypass in a porcine model. Interact. Cardiovasc. Thorac. Surg..

[14] Moody T.W., Ramos-Alvarez I., Moreno P., Mantey S.A., Ridnour L., Wink D. (2017). Endothelin causes transactivation of the EGFR and HER2 in non-small cell lung cancer cells. Peptides.

[15] Mckenzie G.A.G., Hinsley E.E., Hunter K., Lambert D.W. (2014). The endothelin axis in head and neck cancer: a promising therapeutic opportunity?. J. Oral Pathol. Med..

[16] Yanagisawa M. The Endothelin system A new target for therapeutic intervention correspondence to [Internet]. (1994) Available from: http://ahajournals.org. 10.1161/01.cir.89.3.1320 8124823

[17] Kuc R.E., Carlebur M., Maguire J.J., Yang P., Long L., Toshner M. (2014). Modulation of endothelin receptors in the failing right ventricle of the heart and vasculature of the lung in human pulmonary arterial hypertension. Life Sci..

[18] Said N., Smith S., Sanchez-Carbayo M., Theodorescu D. (2011). Tumor endothelin-1 enhances metastatic colonization of the lung in mouse xenograft models of bladder cancer. J. Clin. Invest..

[19] Irani S., Salajegheh A., Smith R.A., Lam A.K.Y. (2014). A review of the profile of endothelin axis in cancer and its management. Crit. Rev. Oncol. Hematol..

[20] Lalich M., McNeel D.G., Wilding G., Liu G. (2007). Endothelin receptor antagonists in cancer therapy. Cancer Invest..

[21] Nelson J., Bagnato A., Battistini B., Nisen P. (2003). The endothelin axis: emerging role in cancer. Nat. Rev. Cancer.

[22] Rosanò L., Bagnato A. (2016). REVIEWS 14th International Conference on Endothelin Physiol Regul Integr Comp Physiol [Internet].

[23] Dang D., Te Y., Aouizerat B.E., Patel Y.K., Viet D.T., Chan K.C. (2020). Targeting the endothelin axis as a therapeutic strategy for oral cancer metastasis and pain. Sci. Rep..

[24] Douglas M.L., Richardson M.M., Nicol D.L. (2004). Endothelin axis expression is markedly different in the two main subtypes of renal cell carcinoma. Cancer.

[25] Liakou P., Tepetes K., Germenis A., Leventaki V., Atsaves V., Patsouris E. (2012). Expression patterns of endothelin-1 and its receptors in colorectal cancer. J. Surg. Oncol..

[26] Tsai K.W., Hu L.Y., Chen T.W., Li S.C., Ho M.R., Yu S.Y. (2015). Emerging role of microRNAs in modulating endothelin-1 expression in gastric cancer. Oncol. Rep..

[27] Dawas K., Loizidou M., Shankar A., Ali H., Taylor I. (1999). Angiogenesis in cancer: the role of endothelin-1. Ann. R. Coll. Surg. Engl..

[28] Hoosein M.M., Dashwood M.R., Dawas K., Ali M., Grant K., Savage F., Lippincott Williams & Wilkins (2007). Altered endothelin receptor subtypes in colorectal cancer. Eur. J. Gastroenterol. Hepatol..

[29] Rosanò L., Spinella F., Bagnato A. (2013). Endothelin 1 in cancer: biological implications and therapeutic opportunities. Nat. Rev Cancer.

[30] Cong N., Li Z., Shao W., Li J., Yu S. (2016). Activation of ETA receptor by endothelin-1 induces hepatocellular carcinoma cell migration and invasion via ERK1/2 and AKT signaling pathways. J. Memb. Biol..

[31] Knowles J.P., Shi-Wen X., ul Haque S, Bhalla A., Dashwood M.R., Yang S. (2012). Endothelin-1 stimulates colon cancer adjacent fibroblasts. Int. J. Cancer.

[32] Smollich M., Wülfing P. (2008). Targeting the endothelin system: novel therapeutic options in gynecological, urological and breast cancers. Expert. Rev. AntiCancer Ther..

[33] Pflug B.R., Zheng H., Udan M.S., D’Antonio J.M., Marshall F.F., Brooks J.D. (2007). Endothelin-1 promotes cell survival in renal cell carcinoma through the ETA receptor. Cancer Lett..

[34] Rosanò L., Salani D., Di Castro V., Spinella F., Natali P.G., Bagnato A. (2002). Endothelin-1 promotes proteolytic activity of ovarian carcinoma. Clin. Sci..

[35] Patel H.J., Olgun N., Lengyel I., Reznik S., Stephani R.A. (2010). Synthesis and pharmacological activity of 1,3,6-trisubstituted-4-oxo-1,4- dihydroquinoline-2-carboxylic acids as selective ETA antagonists. Bioorg. Med. Chem. Lett..

[36] Dai M., Freeman B., Bruno F.P., Shikani H.J., Tanowitz H.B., Weiss L.M. (2012). The novel ETA receptor antagonist HJP-272 prevents cerebral microvascular hemorrhage in cerebral malaria and synergistically improves survival in combination with an artemisinin derivative. Life Sci..

[37] Patel S., Liu X., Liu M., Stephani R., Patel H., Cantor J. (2014). HJP272, A novel endothelin receptor antagonist, attenuates lipopolysaccharide-induced acute lung injury in hamsters. Lung.

[38] Liu X., Khadtare N., Patel H., Stephani R., Cantor J. (2018). Transient blockade of Endothelin-1 mitigates amiodarone-induced pulmonary fibrosis. Lung.

[39] Liu X., Khadtare N., Patel H., Stephani R., Cantor J. (2017). Time-dependent effects of HJP272, an endothelin receptor antagonist, in bleomycin-induced pulmonary fibrosis. Pulm. Pharmacol. Ther..

[40] Olgun N.S., Patel H.J., Stephani R., Lengyel I., Reznik S.E. (2010). Blockade of endothelin-1 with a novel series of 1,3,6-trisubstituted-2- carboxy-quinol-4-ones controls infection-associated preterm birth. Am. J. Path..

[41] Smollich M., Wülfing P. (2008). Targeting the endothelin system: novel therapeutic options in gynecological, urological and breast cancers. Expert. Rev. AntiCancer Ther..

[42] Bhalla A., Haque S., Taylor I., Winslet M., Loizidou M. (2009). Endothelin receptor antagonism and cancer. Eur. J. Clin. Invest..

[43] Rubin S.M., Sage J., Skotheim J.M. (2020). Integrating old and new paradigms of G1/S control. Mol. Cell.

[44] Kent L.N., Leone G. (2019). The broken cycle: E2F dysfunction in cancer. Nat. Rev. Cancer.

[45] Gong W., Wang L., Zheng Z., Chen W., Du P., Zhao H. (2020). Cyclin-dependent kinase 6 (CDK6) is a candidate diagnostic biomarker for early non-small cell lung cancer. Trans. Cancer Res..

[46] Koul H.K., Pal M., Koul S. (2013). Role of p38 MAP kinase signal transduction in solid tumors. Genes. Cancer.

[47] Song X., Wei C., Li X. (2021). The potential role and status of IL-17 family cytokines in breast cancer. Int. Immunopharmacol..

[48] Brix N., Samaga D., Hennel R., Gehr K., Zitzelsberger H., Lauber K. (2020). The clonogenic assay: robustness of plating efficiency-based analysis is strongly compromised by cellular cooperation. Rad. Oncol..

[49] Franken N.A.P., Rodermond H.M., Stap J., Haveman J., van Bree C. (2006). Clonogenic assay of cells *in vitro*. Nat. Protoc..

[50] Grant K., Loizidou M., Taylor I. (2003). Endothelin-I: a multifunctional molecule in cancer. Brit. J. Cancer.

[51] Del Bufalo D., Castro V.D.I, Biroccio A., Varmi M., Salani D., Rosan L., et al. Endothelin-1 protects ovarian carcinoma cells against paclitaxel-induced apoptosis: Requirement for Akt activation [Internet]. 2002. Available from: http://molpharm.aspetjournals.org. 10.1124/mol.61.3.524 11854432

[52] Nelson J.B., Udan M.S., Guruli G., Pflug B.R. (2005). Endothelin-1 inhibits apoptosis in prostate cancer. Neoplasia.

[53] Fennema E., Rivron N., Rouwkema J., van Blitterswijk C., De Boer J. (2013). Spheroid culture as a tool for creating 3D complex tissues. Trends Biotech..

[54] Herrmann D., Conway J.R.W., Vennin C., Magenau A., Hughes W.E., Morton J.P. (2014). Three-dimensional cancer models mimic cell-matrix interactions in the tumour microenvironment. Carcinogenesis.

[55] Popova N.V., Jücker M. (2022). The functional role of extracellular matrix proteins in cancer. Cancers.

[56] Kappes L., Amer R.L., Sommerlatte S., Bashir G., Plattfaut C., Gieseler F. (2020). Ambrisentan, an endothelin receptor type A-selective antagonist, inhibits cancer cell migration, invasion, and metastasis. Sci. Rep..

[57] Tocci P., Blandino G., Bagnato A. (2021). YAP and endothelin-1 signaling: an emerging alliance in cancer. J. Exp. Clin. Cancer Res..

[58] Enevoldsen F.C., Sahana J., Wehland M., Grimm D., Infanger M., Krüger M. (2020). Endothelin receptor antagonists: status quo and future perspectives for targeted therapy. J. Clin. Med..

[59] Slominski R.M., Raman C., Chen J.Y. (2023). Slominski AT. How cancer hijacks the body’s homeostasis through the neuroendocrine system. Trends Neurosci..

