# Supplementary figures and images for: HJP 272, an endothelin receptor antagonist, and its role in cancer cell migration and invasion
Source: Transl Oncol. 2025 Aug 5;60:102492. doi: 10.1016/j.tranon.2025.102492 (PMC12345342; doi:10.1016/j.tranon.2025.102492)

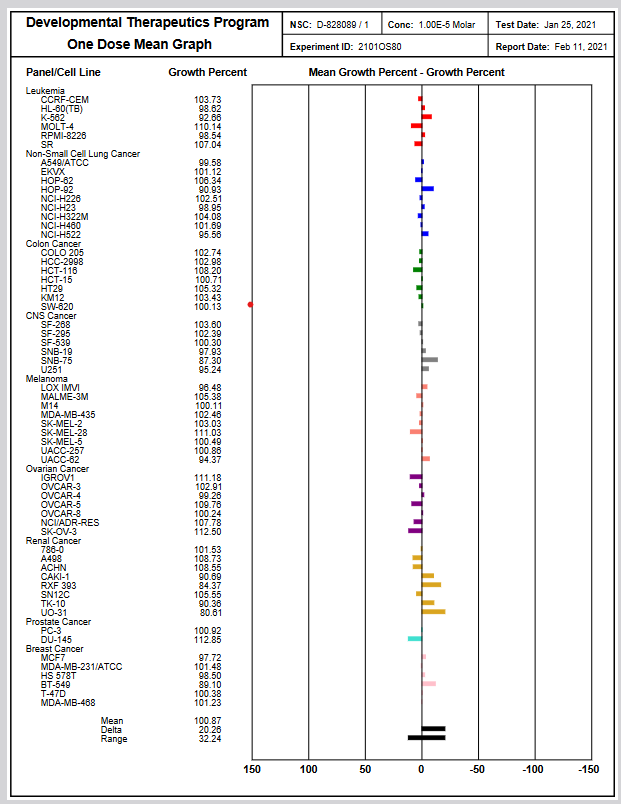


**Supplementary Fig. 1**. NCI-60 data representing one dose (10-5M) mean graph with HJP 272 in the 60-cell line panel.

Supplement: Supplementary file 1 [file mmc1.docx]
