# Supplementary material for: HJP 272, an endothelin receptor antagonist, and its role in cancer cell migration and invasion
Source: Transl Oncol. 2025 Aug 5;60:102492. doi: 10.1016/j.tranon.2025.102492 (PMC12345342; doi:10.1016/j.tranon.2025.102492)
Supplement: Supplementary file 2 [file mmc2.docx]

**
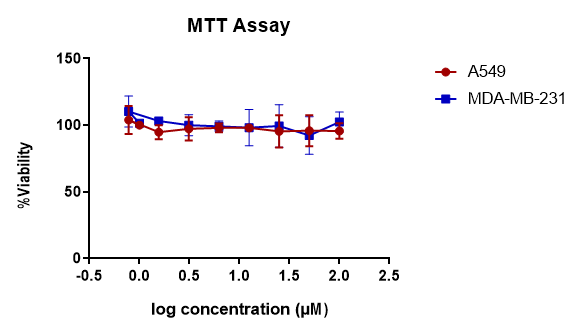
**

**Supplementary Fig. 2.**  Cell viability assay of A549 and MDA-MB-231 cells with HJP 272. A549 and MDA-MB-231 cells were cultured and seeded into 96-well plates at a cell density of 2500 cells/well. Following 24 h of incubation for cell attachment, cells were treated with different concentrations of HJP 272 ranging from 0.75 µM to 100 µM. After 72 h of treatment, 100 µL of 5 mg/mL MTT solution were added to each well and the plates were then incubated at 37^0^C for 2 h. The incubation was followed by aspirating the supernatants and adding 100 µL of DMSO to each well to dissolve the formazan crystals formed due to the reduction of MTT in the viable cells. The optical density (OD) was then measured at 570 nm using a BioTek^TM^ Synergy H1 microplate reader (Winooski, VT, USA). The graph was plotted using GraphPad Prism.
