# Supplementary material for: HJP 272, an endothelin receptor antagonist, and its role in cancer cell migration and invasion
Source: Transl Oncol. 2025 Aug 5;60:102492. doi: 10.1016/j.tranon.2025.102492 (PMC12345342; doi:10.1016/j.tranon.2025.102492)
Supplement: Supplementary file 3 [file mmc3.docx]

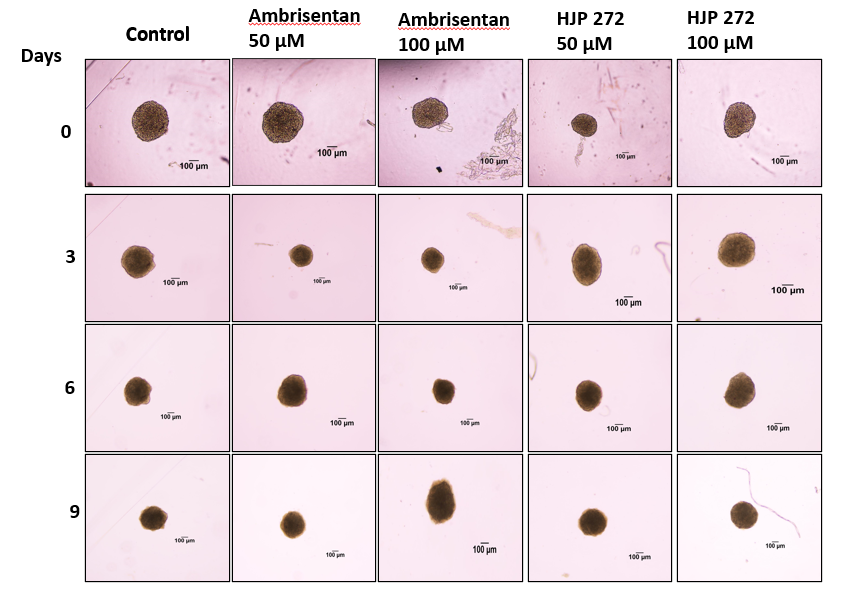


A.

**
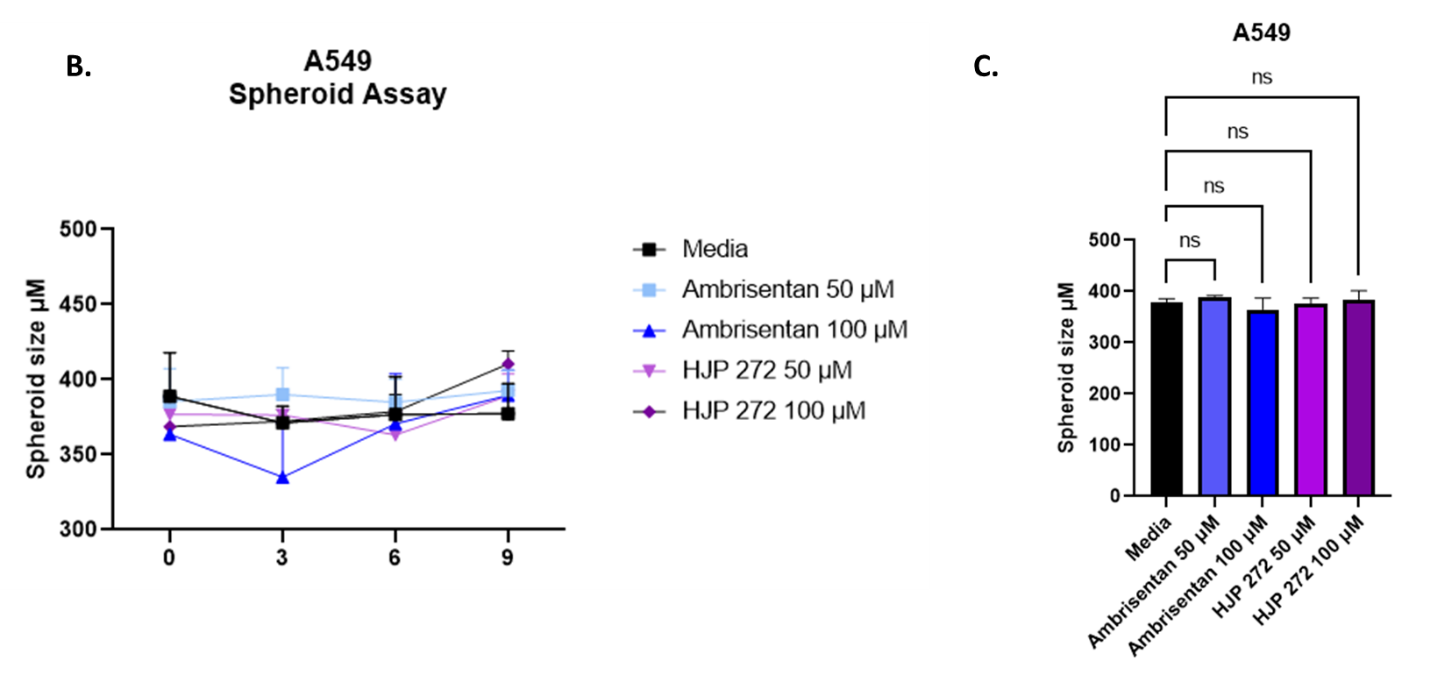
**

**Supplementary Fig. 3**. HJP 272 does not affect A549 cell growth. (A) A549 cells were seeded in 96-well, Nunclon Sphera-Treated, U-Shaped-Bottom Microplates at 1500 cells/well, centrifuged at 1000 rpm for 5 min and incubated at 37^o^C overnight to achieve 3D spheroids. The spheroids were then treated every 72 h with either HJP 272 or Ambrisentan at 50 or 100 µM. The spheroids were imaged every 3 days using a Nikon Eclipse Ts2R-FL camera. (B) Plot of A549 spheroid size over 9 days with or without HJP 272 or Ambrisentan treatments. (C) Histogram showing spheroid sizes with or without HJP 272 or Ambrisentan treatments at the end of 9 days.
